# Electrochemical biosensors: perspective on functional nanomaterials for on-site analysis

**DOI:** 10.1186/s40824-019-0181-y

**Published:** 2020-02-04

**Authors:** Il-Hoon Cho, Dong Hyung Kim, Sangsoo Park

**Affiliations:** 1 Department of Biomedical Laboratory Science, College of Health Science, Eulji University, Seongnam, 13135 Republic of Korea; 2 Division of Advanced Instrumentation Institute, Korea Research Institute of Standards and Science (KRISS), 267 Gajeong-Ro, Yuseong-Gu, Daejeon, 34113 Republic of Korea; 3 Department of Biomedical Engineering, College of Health Science, Eulji University, Seongnam, 13135 Republic of Korea

## Abstract

**Background:**

The electrochemical biosensor is one of the typical sensing devices based on transducing the biochemical events to electrical signals. In this type of sensor, an electrode is a key component that is employed as a solid support for immobilization of biomolecules and electron movement. Thanks to numerous nanomaterials that possess the large surface area, synergic effects are enabled by improving loading capacity and the mass transport of reactants for achieving high performance in terms of analytical sensitivity.

**Main body:**

We categorized the current electrochemical biosensors into two groups, carbon-based (carbon nanotubes and graphene) and non-carbon-based nanomaterials (metallic and silica nanoparticles, nanowire, and indium tin oxide, organic materials). The carbon allotropes can be employed as an electrode and supporting scaffolds due to their large active surface area as well as an effective electron transfer rate. We also discussed the non-carbon nanomaterials that are used as alternative supporting components of the electrode for improving the electrochemical properties of biosensors.

**Conclusion:**

Although several functional nanomaterials have provided the innovative solid substrate for high performances, developing on-site version of biosensor that meets enough sensitivity along with high reproducibility still remains a challenge. In particular, the matrix interference from real samples which seriously affects the biomolecular interaction still remains the most critical issues that need to be solved for practical aspect in the electrochemical biosensor.

## Background

The electrochemical biosensor is the analytical devices that transduce biochemical events such as enzyme-substrate reaction and antigen-antibody interaction to electrical signals (e.g., current, voltage, impedance, etc.) [1, 2]. Since Clark developed the 1st version of electrochemical biosensor for blood glucose, various types of biosensor have consecutively been introduced and commercialized for diverse applications [3]. In this electrochemical biosensor, an electrode is a key component, which is employed as a solid support for immobilization of biomolecules (enzyme, antibody and nucleic acid) and electron movement. Various chemical modification methods are applied for this purpose via amine- and carboxyl (1-ethyl-3-(3-dimethylaminopropyl)carbodiimide: EDC), aldehyde- (hydrazide) and thiol (maleimide), depending on the chemical groups on the electrode in the presence of or absence of supporting materials [4–6]. Since inappropriate immobilization may cause loss of activity, less specificity, and low biocompatibility, it is crucial not only to maintain orientation and biological activity of the biomolecules upon immobilization. In addition, employing proper functional material for the electrode is a key process for the high performance of biosensors.

Recently, various electrochemistry-driven biosensing methods have been introduced for simple and miniaturized analytical devices for on-site analysis. This trend can be applied to replace the commercial lab instruments manufactured by the renowned in vitro diagnosis (IVD) companies which claim high sensitive measurement of analytes and automation. However, developing an ideal on-site version of the biosensor to meet a required sensitivity along with high reproducibility still remains a challenge. Employing functional nanomaterials used as a supporting matrix for signal enhancement has gained attention for high-performance electrochemical analysis [7]. Nanomaterials endow the large surface area, enabling support increased loading capacity and the mass transport of reactants, which results in a synergic influence for signal amplification [8].

**Fig. 1:**
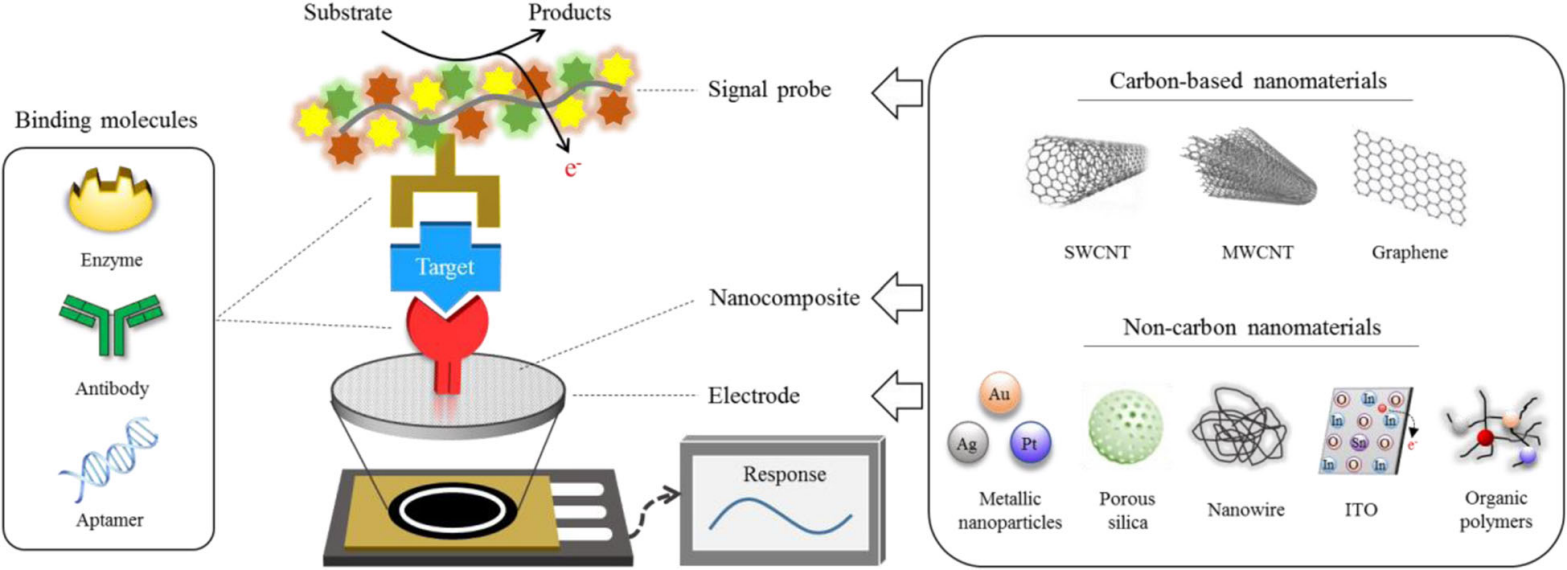
Scheme of analytical principle for electrochemical biosensors based on carbon and non-carbon nanomaterials

Here, we primarily focus on the functional nanomaterials (carbon-based and non-carbon-based) which were employed in the diverse forms of electrochemical biosensor for improving an analytical performance in terms of sensitivity as shown in Fig. 1. The nanomaterials employed as electrodes or assisting matrices should meet the following requirements for signal enhancement: assisting electro-catalytic property, outstanding electron movement capability and great biocompatibility with capture biomolecules. The nanomaterial incorporating electrochemical strategies can be applied for both a paper and a microfluidic type of biosensor applications, which are practical sensing platforms for point-of-care version of biomolecular detection.

### Carbon-based nanomaterials

Carbon-based nanomaterials are very useful and have been applied to diverse industrial fields [9]. Here, we introduce the current electrochemical biosensors employing carbon nanomaterials, e.g., carbon nanotube (CNT) and graphene for analytical improvement (Table 1). CNTs can be used as an electrode structure because of their extraordinary mechanical stability, large surface area, and remarkable electrical conductivity caused by orbital hybridization (*sp*^*2*^ type) between adjacent carbon atoms [10]. There are two types of CNTs: single-walled and multi-walled carbon nanotube. Graphene, a 2-dimensional hexagonal pattern of carbon atoms, can also be adopted as an electrode due to its higher specific surface area than CNTs [5]. However, graphene has a low throughput and hydrophobicity, which limits its usability in biosensor applications [11]. Graphene oxide (GO) and reduced graphene oxide (rGO) solved the problems by increasing hydrophilicity of the graphene layer and eliminating the oxygen groups of GO, achieving an extraordinary electrical conductivity and ease of surface modification for immobilization of biomolecules [12].

**Table 1:** Summary of representative carbon-based nanomaterials used in electrode and label of electrochemical biosensor

Materials	Advantage	Limitations	Feature	Limit of detection	Ref.
SWCNT	Large surface area to volume ratio (S/V) Low charge-carried density Delocalized π-orbitals Electrical conductivity improvements	Limited surface to interface with large biological components Nonspecific adsorption of protein Difficult manipulation during sensor fabrication process Difficult chemical functionalization	Electrode	DeoxyriboNucleic acid (DNA) 71 pM	[13]
			Electrode	Glucose 7.06 μA/mM	[14]
			Electrode	aflatoxin B1 (AFB1) 0.01 nM	[15]
			Electrode	Anti-IgG 0.2 pM	[16]
MWCNT	Excellent conducting and electro-catalytic properties	Need to functionalize surface for increasing biocompatibility Irreversible agglomerates in aqueous solution	Electrode	Carcinoembryonic antigen (CEA) 0.0055 fM	[17]
			Electrode	Transforming growth factor beta 1 (TGF-β1) 0.05 pM	[9]
			Electrode	Prostate specific antigen (PSA) 0.11 fM	[18]
			Electrode	Mouse IgG 0.066 pM	[19]
			Label	PSA 0.13 pM	[20]
Graphene	High S/V Large active sites Fast electron transfer High thermal conductivity Better mechanical flexibility Good biocompatibility	Hard to dissolve in water	Electrode	dibutyl phthalate (DBP) 0.025 μM	[21]
			Electrode	PSA 0.33 pM	[22]
			Electrode	Cystatin C 0.002 nM	[23]
			Label	Cry1C 0.02 pM	[24]
			Label	CEA 0.003 pM	[25]

### Single-wall carbon nanotubes

Recently, single-walled carbon nanotubes (SWCNTs) commonly used in biosensor to enhance electrical properties. SWCNTs have great electronic and mechanical characteristics [26]. Due to its physicochemical properties, the SWCNTs have gained great attention in electrochemical biosensors [27]. Its huge surface area could increase the quantity of the immobilized enzymes, widen the reaction areas between the enzyme and the substrate, facilitate electrical conductivity and increase the signal response of the biosensors [15]. All these properties claim that SWCNTs could be capable of stimulating electron-transfer reactions for several biological molecules. However, the insolubility of SWCNTs may be problematic in biological applications. In order to overcome the stable insolubility in aqueous solutions, some nanocomposites with unique biocompatibility properties have been adopted with SWCNT [28, 29]. In addition, polymer nanocomposite incorporating carbon nanotubes is electrically conductive when the content of the filler is higher than the critical level [7].

Mao et al. described a simple, label-free electrochemical impedance spectroscopic technique for sequencing-specific DNA detection using SWCNTs as support for the DNA probe [30]. The SWCNTs are incorporated into gold electrodes with surface modification by self-assembled monolayers made of thiol derivatives. The single-stranded DNA probe is anchored to the SWCNTs support via covalent bonding between the -COOH in the nanotubes and the -NH_2_ groups at the 5 ‘end of the ssDNA. They claim that SWCNTs as supporting matrix for probe DNA significantly increase the surface loading capacity on the electrode surface and therefore significantly lower the detection limit of target DNA. The SWCNT and polypyrrole multilayer film on platinum, which is coated from polyvinylidene fluoride membrane was manufactured by Shirsat et al. [14], demonstrating the feasibility of SWCNT-Polypyrrole multilayer biosensor for glucose monitoring. Here, a layer by layer form of polypyrrole and SWCNT provided a useful solid matrix for enzyme immobilization, indicating a good performance with excellent linearity from 1 mM to 50 mM of glucose concentration, and high sensitivity of 7.06 uA/mM. In addition, an electrochemical sensor implemented on SWCNT combining screen printed electrodes [13] and glassy carbon electrode (GCE) [31] were also introduced for better performances.

Multi-functional SWCNTs provide a basis for novel biosensor systems incorporating the immobilization of distinct biomolecules in combination with enzymes and redox mediators. Multiple functionalizations enable the co-immobilization of biomolecules and rigid spacers between SWCNTs to enhance the mechanical solidity of SWCNT structures. Holzinger et al. researched multiple functionalizations of SWCNTs to create a polyvalent sensing electrode [32]. Three separate pyrene compounds are simultaneously immobilized on the surface of the nanotube with π-stacking a single-step process of basic dip coating.

SWCNTs can also be used as an electrochemical mediator. Jiang et al. published a feasibility study for the terminal protection of small-molecule-linked DNA [33]. Due to the fact that the SWCNTs adsorbed on the insulating 16-mercaptohexadecanoic acid self-assembled monolayer could efficiently mediate electron transfer between the electrode and electron mediator such as ferrocene carboxylic acid, a strong redox current was detected. Gutierrez et al. reported an electrochemical quantification detection for Cd (II) combined with a glass carbon electrode by dispersion of SWCNTs functionalized with cysteine [31]. The functionalization of SWCNTs was achieved by the reaction between the carboxylic groups of oxidized SWCNT and the amino groups of S-triphenylmethyl cysteine using a benzotriazole-based binding chemistry agent to activate carboxylic residues. Multi-functional SWCNTs provide the platform for new biological systems involving immobilization of different biomolecules exhibiting complementary activities or associations at the molecular level of enzymes and redox mediators.

As mentioned, SWCNTs have drawn tremendous interest due to their great mechanical, chemical and optoelectronic traits, making them attractive nanomaterial for various applications [34]. However, the as-produced SWCNTs have a wide distribution of different chiral species with different characteristics (e.g., electronic structures). Highly filtered and well-separated process is very crucial to take full advantage of the SWCNTs [34]. Furthermore, SWCNT-based biosensors have also restricted surface area to interact with large biorecognition agents like mammalian cells, control the sensor manufacturing process, and undergo chemical modification [10]. In particular, the non-specific protein adsorption on nanotubes is not desirable, particularly when using biological fluid samples containing several co-existing proteins and lipids [35]. Therefore, more advanced sensors are required to address the practical issues related to blockage and unintended disruption induced by a non-specific binding effect that directly affects the analytical performances (selectivity and sensitivity) of biosensor [10].

### Multi-wall carbon nanotubes

MWCNT comprises multiple layers of concentric single-walled graphene cylinders of which structure is supported via Van der Waals forces with an interlayer spacing of 3.4 Å [36]. The MWCNTs have a sidewall structure similar to the graphite basal plane [37]. As a consequence, electron transfer speeds may be comparable to the graphite edge-plane electrode. While MWCNTs are still known as a 1-dimensional form of carbon, the unique properties present inside single-walled and double-walled carbon nanotubes are not as prominent. Nevertheless, the MWCNTs with excellent conduction and electro-catalytic characteristics have also been employed as a modified scaffold on the electrode (Table 1). The solid substrate for antibody immobilization can be changed by chemical treatment [36].

As significant structural degradation of MWCNTs occurred during functionalization, including decapping at the end of the tubes and slicing and breaking the length of MWCNTs, proper functioning of the MWCNT surface is critical. Oxygen-functionalized multi-walled carbon nanotube (f-MWCNT) has been provided for stable immobilization by covalent bonding between the oxygen functional groups of f-MWCNT and -NH_2_ groups of the antibody [38]. Zheng and Zheng described that the immobilization of the non-polar amino acid chain of gelatin at the side wall of MWCNT takes place by hydrophobic - hydrophobic forces, leading to a stable dispersion of MWCNT [39]. Also, other similar studies indicate that the strength of the MWCNT/gelatin dispersion could be remarkably improved by increasing the amount of MWCNT [40, 41]. Viswanathan et al. reported a disposable electrochemical biosensor with polyethyleneimine wrapped MWCNT screen-printed electrode [17]. The positively charged polyethyleneimine chains were ionically wrapped on the surface of carboxylic acid-modified MWCNT.

Numerous enzyme biosensors have introduced CNT modified electrodes as a multifunctional scaffold. Nonetheless, the CNT-modified electrode biosensors have been documented to a much smaller extent. This is likely because immobilization techniques for this altered electrode depend mostly on direct adsorption or covalent linkages that may reduce the stability of biomolecules and the reproducibility of bioelectrodes. Anchez-Tirado et al. used MWCNTs as an electrode modifier for the development of a transforming growth factor - β1 (TGF-β1) immunosensor, involving the reaction of Cu(I)-catalyzed azide-alkyne cycloaddition to synthesize alkyne-azide conjugates [9].

Nanoparticle incorporating MWCNTs can be an alternative method to improve analytical performances. Mingdang Li et al. introduced an ultrasensitive electrochemical biosensor employing the AuNPs hybrid MWCNTs-SO_3_H as electrode material which was coupled mostly by the physisorption [18]. Xu et al. developed an amperometric glucose biosensor by means of alternating electrostatic self-assembly of glucose oxidase and dendrimer-encapsulated Pt nanoparticles on MWCNTs [42]. Here, the outstanding electrocatalytic behavior and the unique 3-D structure of the enzyme electrode resulted in a low limit of detection with wide linearity, great precision and improved operational stability.

One of the main drawbacks of CNTs is that their manufacturing process is not fully controlled. Aggregation and low uniformity are critical issues [43]. Furthermore, CNTs are typically insoluble, hindering their practical approaches. The MWCNTs in aqueous solution tends to form irreversible aggregation phenomenon by strong π -π stacking and van der Waals forces, which severely restrict their use [18, 43]. To this end, the MWCNT surfaces undergo chemical modification with sulfonic acid groups, hydroxyl groups and carboxyl groups for increasing dispersity and uniformity of film on the electrode surfaces [44–46].

### Graphene

Graphene in the shape of a 2-D hexagonal lattice form has received considerable attention [10]. Graphene has been employed in various areas of the biosensor, in particular for electrochemical sensing platforms. Graphene has the same intrinsic physicochemical characteristics as graphite and CNT, including large surface area and multiple functional sites. As shown in Table 1, it is preferable to other carbon-based nanomaterials on the basis of the following physicochemical properties: exceptional electron transfer, improved thermal conductivity, mechanical stability and biocompatibility [47]. However, it is difficult for graphene to dissolve in water and therefore its surface should be modified with hydrophilic functional groups such as -COOH [47, 48]. This method facilitates improved solubility and molecular immobilization by known NH_2_-COOH chemistry aided by 1-ethyl-3-(3-dimethylaminopropyl) carbodiimide (EDC). Also, reduced graphene (rGO) can also be used as a supporting solid substrate for ease of surface modification [10]. However, improving its reproducibility and reliability is a critical challenge for its high-performance analysis. With more sophisticated manufacturing methods, graphene is widely employed as an alternative to traditional electrode used in the electrochemical biosensor.

Graphene has been considered as the ideal support source for label-free biosensor due to its excellent electronic and mechanical properties [5]. Integrating metallic nanoparticles on highly conductive surfaces is desirable for the manufacturing electrode owing to its huge surface area, electrical conductivity and enzyme immobilization capacity [5, 49]. In particular, chemically modified graphene contains numerous defects/vacancies and possesses functional groups, thereby acting as a highly desirable solid support for the immobilization of inorganic nanoparticles as well as enzymes with improved stability and loading efficiency. Han et al. introduced a label-free biosensor, which was manufactured with rGO/AgNP composites supporting material. Small AgNPs are more capable of improving the electrical characteristics of rGO than large AgNPs [50]. A small molecule, e.g., sodium citrate, and a commonly used reducing agent, were utilized to produce rGO/AgNP composites with improved electrical conductivity [22].

Wu et al. reported gold nanoparticle dotted reduced graphene oxide (rGO-AuNP) which was used as a substrate for an aptamer biosensor [51]. Wang et al. reported a responsive acetylcholinesterase biosensor in combination with a screen-printed electrode modified with iron oxide nanoparticle [52]. Since the following advantages: large active surface area, effective electron transfer and affinity of iron oxide for the phosphoric band, the nanocomposite film has provided numerous active sites and microenvironments to facilitate reaction acetylcholinesterase and sustain enzymatic activity.

The redox substrate, which facilitates a strong current signal, has paved the way to enhance the analytical performance [53]. GO was used to increase the electrical conductivity on the surface and the stability of the 3-D porous structure, resulting in dual signal enhancement [54]. Wang et al. studied a label-free electrochemical biosensor for the quantitative detection of alpha-fetoprotein [55]. Multifunctionalized graphene (TB-Au-Fe_3_O_4_-rGO) was used to change the electrode surfaces to achieve the signal amplification of the electrochemical signal. Here, as a type of redox probe, toluidine blue (TB) produce the electrochemical signal. Trindade et al. developed an electrochemical sensor with intrinsic redox behavior mediated by ferrocene for Cystatin C, an early renal failure biomarker, on a functionalized graphene base [23]. The current response was mediated by GO-ferrocene nanofilm with redox activity from surface-confined electroactive species.

Nanocomposite and nanohybrid can often show improved physicochemical properties [56–59]. A novel two-dimensional all-carbon nanocomposite electrode platform on the basis of ordered mesoporous carbon and fullerene can significantly accelerate the electron transfer rate and provide effective electrochemical sensing [60]. All carbon nanotubes and graphene are very suitable for electroanalysis [61, 62]. Therefore, the fusion of 1-D CNTs with 2-D graphene and the use of nanohybrid carbon for electrochemical determination is worthwhile. For example, SWCNTs–graphene nanosheet hybrid films [56] and highly packed graphene–CNT films as electrodes for aqueous supercapacitors with the high volumetric performance [63] were introduced. Cheemalapati et al. reported simultaneous electrochemical determination using MWCNT/GO nanocomposite-assisted glassy carbon electrode [64]. Higher electrocatalytic activity relative to either pure MWCNT or GO is caused by synergistic effects between MWCNT and GO.

### Non-carbon nanomaterials

Recently, non-carbon nanomaterials have been employed as alternative supporting components of the electrode for improving the electrochemical properties of biosensors. In this section, we introduce various types of nanomaterials and classified into five categories, metallic and silica nanoparticle, nanowire, and indium tin oxide (ITO), organic polymers, which were used as a nanostructural electrode or supplementary components (Table 2). The properties of metallic nanoparticles (MNPs) provide a large surface area for improving immobilization efficiency of biomolecules and show unique abilities for electron transfer, catalytic activity, and great biocompatibility [65, 66]. The silica nanoparticle also offers several benefits such as excellent uniformity and tunable pore structure, high surface-to-volume ratio, and chemical-modifiable surface [67]. The nanowires as a one-dimensional structure have excellent potential due to their high width-to-length ratio, small size and electronic characteristics in the bio-sensing approaches [68, 69]. The unique properties can be applied to improve electrical conductance by synthesizing different compounds, resulting in high electron transfer efficiency. The ITO is one of the most commonly used conductive materials for electrochemical biosensing owing to its excellent electrical conductivity and low price [70]. Furthermore, ITO electrodes can be used to enhance electroanalytical activity through the method of surface modification using nanomaterials that provide large surface area, biorecognition matrix, electrochemical reaction catalyst and electron transfer enhancers [71]. The organic polymers which are easily processible and printable on diverse solid substrates were applied to fabricate the nanomaterials composing an electrode and signal probe of the electrochemical biosensor. The hybrid materials can provide several merits for application in biochemical detections via unique properties of the polymer, such as solubility, functionality, and flexibility [72].

**Table 2:** Summary for the features of non-carbon nanomaterials to construct electrochemical biosensors

Materials	Advantage	Limitations	Feature	Limit of detection	Ref.
Metallic nanoparticles	Efficient electron transfer Increase in S/V Supplying superior conductivity Good biocompatibility Easy functionalization	Electrical instability in high salt concentration Inconsistent upon signal amplification	Electrode (AuNPs)	CEA 0.01 pM	[65]
			Electrode (AgNPs)	PSA 0.1 pM	[73]
			Label (Fe3O4/Ag/Au)	IgG 0.33 fM	[74]
			Label (PtNPs)	Alpha-fetoprotein (AFP) 0.001 pM	[66]
			Label (Pt/Cu NPs)	PSA 0.55 fM	[75]
Mesoporous silica nanoparticles (MSN)	High pore volume and surface area Good electron transfer and high loading capability Well-defined surface properties Tunability of size and shape	Difficult in preparation of well-ordered Scattered size distribution Formulation of stable-colloidal suspensions	Label (MSN/Au)	PSA 0.01 pM	[76]
			Label (MSN/Au/Ru)	p53 22.8 fM	[77]
			Label (MSN/Ag)	N6-methyladenosine (m6A) 0.078 nM	[78]
Nanowire	High S/V Rapid response High electro-catalytic capability and reproducibility Improvement of the charge transfer and stability	Decrease in electrostatic potential with distance	Electrode (Ag)	IgG 0.03 pM	[67]
			Label (Pt)	hepatitis B surface antigen (HBsAg) 0.14 fM	[79]
			Electrode (Cu_2_O)	AFP 0.0015 fM	[80]
			Electrode (Si)	cardiac troponin I (cTnI) 0.14 pM	[81]
ITO	Low cost / High transmittance Good electrical conductivity Ease of surface modification	Slow kinetics of electron- transfer upon coating surface with antibodies	Electrode (ITO/PET)	Receptor for Activated C Kinase 1 0.83 fM	[82]
			Electrode (ITO)	Creatine kinase-MB (CK-MB) 0.24 fM	[83]
			Electrode (ITO/Au)	Guanine 250 nM	[84]
			Electrode (ITO)	microRNA 2.0 fM	[85]
Organic polymer	High-throughput Low-cost Good flexibility, functionality, solubility, and specificity	Need to reproducibility of thin-film morphology Chance to physical delamination High operating voltages Uncertainties in material stability	Electrode (5, 2′:5′,2″-terthiophene-3′-carboxylic acid)	Glutamate 0.1 μM	[86]
			Electrode (polypyrrole)	Serotonin 0.03 μM	[87]
			Electrode (poly(3,4-ethylene dioxythiophene)	Dopamine 0.22 μM	[88]

### Metallic nanoparticles

Metallic nanoparticles were also used as solid support for electrochemical biosensors, improving not only the transfer efficiency but also the surface-to-volume ratio (Table 2) [10]. Gold nanoparticles (AuNPs) are the most commonly used metal nanoparticles in electrochemical biosensors owing to their unusual biocompatibility and easy protein functionality. In order to obtain a good signal-to-background ratio, the electrical signal should be strong when the gold tag is precisely bound to the sensing electrode. For this reason, it is possible to use a low electrocatalytic electrode where most redox reactions are slow except for the redox reaction of the mediator. Lin et al. documented a signal enhancement method involving the mounting of many AuNPs on microbead poly (styrene-co-acrylic acid) as microbeads by in situ tracing tag synthesis [89]. Here, AuNPs are triggered to silver metallization with silver nanoparticles (AgNPs), which could be easily detected by an anodic stripping analysis. Ding et al. produced a new impedimetric immunosensing technique for sensitive prostate-specific antigen using AuNP-decorated nanosheets that were used to tag anti-PSA antibody and horseradish peroxidase [90]. Fang et al. reported ultrasensitive and incubation-free electrochemical biosensor using a gold-nanocatalyst label mediating outer-sphere-response-philic and inner-sphere-response-philic species [83], claiming the sensitive measurement of creatine kinase – muscle brain (CK-MB).

AuNPs can also be used to form electrodes owing to their great electrochemical behavior resulting from intrinsic metallic characteristics, where free electrons migrate from the valence to the conduction band. Cai et al. described a ratiometric electrochemical method that was manufactured using Polythionine – Gold (PTh – Au) as an electrode [65]. The immunosensor could detect analyte with good specificity within a wide linear range with a detection limit of 2.2 pg/mL.

Hybrid electrodes employing gold nanoparticles in combination with other materials such as silicon oxide, carbon nanosphere, and calcium carbonate have recently been studied to enhance the synergistic effects influencing analytical performances [91–93]. Gold catalysts for inert oxide support such as silicon dioxide (SiO_2_) must be prepared in a highly dispersed condition [94]. Improved catalytic activity may occur from geometrical effects associated with imperfect sites such as kinks, steps or edges or from electrical effects resulting from differences in the density of small gold particles [94–97].

When metallic nanoparticles are incorporated with carbon nanotubes, the final composite can be further improved [98, 99]. The use of AuNP – CNT nanocomposites provides many benefits, such as simple surface alteration, exceptional electrical conductivity and high sensitivity and selectivity by their ability to separate the oxidation potential of specific analytes [100–102]. In this regard, a variety of metal nanoparticle/carbon nanotube hybrid nanostructures can be applied as sensitive modified electrodes [103]. Calcium carbonate (CaCO_3_), a natural mineral with good biocompatibility, has been shown to improve enzyme efficiency [104]. Spherical polymorph is supposed to be used for a variety of purposes owing to its specific characteristics, such as larger surface area better water dispersity and lower specific gravity than the other crystal models [105, 106]. Gold nanoparticles stabilized via sodium citrate were assembled on the surface of porous carbonate microspheres to prepare the hybrid product. It ensures biocompatibility and better solubility and water dispersibility [105].

Among different noble metal nanoparticles (e.g., silver, copper, platinum, etc.), Silver nanoparticles (AgNPs) are subject to new engineering techniques with highly novel resulting morphologies and traits. Such NPs have several advantages, making it easier to pass electrons more effectively and to accommodate more active sites on their surface [107, 108]. Nevertheless, the use of metallic nanoparticles alone is not adequate for high sensitive detection, which inevitably involves more comprehensive methods. Past work demonstrates that the electrical conductivity of reduced graphene oxide can be dramatically enhanced by the addition of silver nanoparticles and the repair of structural defects of the reduced graphene oxide [10].

Recently, Fe_3_O_4_ magnetic nanoparticles have received interests in various research fields, such as biotechnology, pharmacy, cell separation and drug delivery, due to their useful properties including biocompatibility, low toxicity, high super magnetism, catalytic action, artificial mimetic activity and fast electron transfer [109, 110]. It would, therefore, be possible to use them in applications [111]. Zhang et al. published a novel non-enzymatic electrochemical sensor using Fe_3_O_4_ nanospheres with Ag@Au nanorods as a nano electrocatalyst [74]. Ag@Au-Fe_3_O_4_ nanohybrid modified electrode presents better electrocatalytic behavior towards the reduction of hydrogen peroxide than Fe_3_O_4_ nanospheres or Ag@Au nanorods owing to the synergetic catalytic influence.

In recent years, Pt nanostructures have been extensively researched in the direct electrocatalytic oxidation of small organic molecules (such as methanol and formic acid) [112]. In most cases, however, the structure of the catalysis is not yet well understood, although many catalysts have been introduced. Over several decades, Pt NPs with a variety of shapes have been studied, including nanocubes [113], nanotubes [114], and dendritic NPs [115]. Tian et al. discovered tetrahexahedral Pt nanocrystals with high index facets and excellent electro-oxidation function [116]. Here, single-crystal surfaces of bulk Pt showed that high-index planes basically presented significantly higher catalytic activity than the most typical stable planes. Wang et al. reported a synthesis of platinum (Pt) nanoparticles with optimized sizes and shapes to improve catalysis to reduce oxygen [113].

Electrical instability is one of the drawbacks of metallic nanoparticles due to its susceptibility to salt concentrations, which may lead to precipitation of aggregation [10]. Appropriate chemical and biological adjustments of the nanoparticle surfaces are therefore important for the use of such nanoparticles in human specimens with a high salt concentration. In contrast, metallic nanoparticle-driven signals are incompatible with signal enhancement, which limits reproducibility [47]. Therefore, quality control of nanoparticles produced from a liquid-phase reaction in combination with a fine-tuning reduction should be considered for better performances.

### Silica nanoparticle

Mesoporous materials with a crystalline structure and a huge surface area received research interest. Through rationally designing and tailoring the nanoscale mesoporous structure, the transport of electrons can be significantly improved (Table 2) [67]. More recently, a series of mesoporous materials have been provided on the basis of a controlled release process for loading and release of cargo can be tuned by specific external stimuli [117].

Fan et al. announced that a controlled release method using mesoporous silica nanoparticles (MSN) with cleavable linkage was designed to create an electrochemical sensor for the quantitative measurement of prostate-specific antigens [76]. Under the acidic environment, the coated Au nanoparticles were removed from MSN-Th (thionine)-Au by hydrolysis of the acid-labile acetal linker resulting in the release of encapsulated Th. Zhou et al. documented a dual-signal electrochemical sensor with a signal probe using mesoporous SiO_2_ coupled with CdSeTe@CdS quantum dots and Ag nanoclusters [118]. Composites are selected as probes, for their individual oxidation peaks without interruption, and for the easy preparation process to achieve a relatively uniform and controlled length. The great dispersion properties and plentiful functional groups also make it easier for Ag NCs and CdSeTe@CdS QDs to be coupled with biomolecules.

### Nanowire

Nanowire has exceptional potential as an alternate sensing technique because of its small scale, high surface-to-volume ratios, and its electrical, optical and magnetic characteristics (Table 2) [68]. Nanowire has been considered to be more versatile and flexible than larger wires. However, its one-dimensional configuration shows a high ratio of width to length, which results in particular physical properties comparable to the quantum phenomena [69]. The electrical conductivity of nanowires can be managed by synthesizing diverse elements and chemical compounds, such as metals (Ni, Cu, Au, Pt, etc.), metal oxides (ZnO, SnO_2_, Fe_2_O_3_), semiconductors (Si, InP, GaN) [10]..

Silver nanowires have extraordinary electrical properties (e.g. rapid response, effective electrocatalytic behavior and reproducibility) and can be used as an active carrier for specific signal generators for multiple electrochemical measurements. Cao et al. reported a silver nanowire-based electrochemical immunosensor [67]. The superconductivity of Ag nanowires in the sensing probe was synthesized by a chemical process with mesoporous ZnO nanostrawberries. Platinum nanowires are biocompatible with biomolecules (proteins and nucleic acids) and have outstanding catalytic properties for the reaction of hydrogen peroxide [79].

It has also been documented that certain metal oxides such as Zn, Fe, Sn, and Ti show the facilitation of electron transfer rate and affect the efficiency of nanowire sensors. In addition, these metal composites allow nanowire surfaces biocompatible and catalytic and improves sensing performances. Wang et al. introduced a TiO_2_ nanowire-based microelectrode for rapid recognition of *Listeria monocytogenes*, which induces outbreaks of food poisoning [119]. The degree of impedance change induced by the antibody-bacteria complex on the nanowire was measured in proportion to the quantity of *Listeria monocytogenes*. Also, cuprous oxide nanowires with good electronic characteristics and electrocatalytic efficiency have become a kind of attractive nanomaterials. Wang et al. used a Cu_2_O nanowire to improve the special electronic, optical and mechanical characteristics of 2-D nanomaterials in label-free electrochemical biosensors [80].

Semiconductor nanowire - silicon nanowire field-effect transistors (Si-NWFETs) has been recognized as a versatile electrical sensing tool owing to its high sensitive, real-time and label-free properties. Kim et al. used the Si-NWFET-based immunosensor to detect cardiac troponin I, which is one of specific biomarkers of acute myocardial infarction [81]. Here, the nanowire was lightly doped on the FETs to improve its electrical sensitivity. The nanowire presents a very sensitive limit of detection as low as 5 pg/mL.

Compared with the metallic nanoparticles and CNTs, the use of polymeric nanomaterials has several merits, including low-temperature synthesis, tunable electrical conductivity and no need for purification, or catalytic deposition step [120]. However, polymeric nanomaterials are typically less suitable as components in biosensor due to their relatively low electrical conductivity than CNTs, as well as their non-oriented nanofiber morphology, resulting in low analytical performances. To resolve these problems, Guan et al. synthesized a nanotube array with conductive polyaniline (PANI) with good molecular alignment and orientation for ultrasensitive DNA identification [120]. PANI has primarily been used as an electron mediator for signal transmission to the electrode, which can be caused by gold nanoparticles.

While nanowire electrochemical measurements have several analytical merits over the traditional methods listed, a major drawback is that the electrostatic potential of the nanowire arising from the charge on the analyte molecule decays exponentially towards zero at a distance (Debye length) [121]. To circumvent the intrinsic problem, a reduction in the size of the capture antibody by fragmentation with a proteolytic enzyme or a single-chain fragment provides technical troubleshooting [122]. Density control of the capture molecules on the electrode may also be known to reduce the inherent limitations of the nanowire.

### Indium tin oxide

ITO has commonly been employed as an electrode thanks to its special optoelectronic characteristics and excellent transmittance (Table 2) [123]. The benefits of ITO are cost-effective and great electrical conductivity [124]. ITO’s hydroxyl groups on the surface can be functionalized with a variety of chemical compounds (e.g. silane derivatives) to provide active surfaces of amines, carboxylic acids, and thiols also referred to as self-assembled monolayers (SAMs) for the capture antibody immobilization.

The point-of-care (POC) version of the ITO-based electrochemical biosensor can be used because of its good electrical conductivity and ease of process to be deposited as a thin film [10]. The glass-like properties enable effective immobilization of biomolecules by surface modification with various functional monolayers such as aldehyde, carboxylic acid, amine, and sulfhydryl. Given its exceptional traits, the ITO-based electrode also has some technical barriers. One of the main drawbacks is that the electron transfer rate of the ITO electrode is much slower than that of the noble metal and carbon-based electrodes. In particular, the negative phenomenon increases when electrodes are coated with biomolecules. Therefore, this problem should be minimized with appropriate surface functionalization of conductive polymers and electron mediators that enhance electron transfer.

### Organic conductive polymer

The fabrication of miniaturized, low-cost, flexible sensors based on organic electronics via high-throughput techniques (e.g. printing) is expected to provide important benefits for applications in chemical and biological detection. The rapid maturation of synthetic methodology in the field of organic electronics has lead to the creation of new materials at an incredible rate and an increased understanding of semiconductor analyte interactions. Owing to these advances, we have seen steady improvements in sensitivity, stability, and specificity, in addition to the detection of a wide range of chemical analytes [72]. These conducting polymers that are easily processible and printable on diverse solid supports have applied to fabricate the transistor and electrode.

Conductive organic polymers have been applied to organic thin-film transistor (OTFT) structure incorporating glucose oxidase (GOX). Here, the diffusion of protons generated by the oxidation of glucose could be improved with the organic polymer, determining the response time of these sensors. Elkington et al. reported the effect of poly (3-hexythiophene) (P3HT) OTFT to detect glucose concentrations in saliva [125]. They also suggested that the OTFP architecture could improve the surface uniformity of deposited films.

The use of polymers such as polyaminobenzoic acid endow carboxyl groups on the ITO surfaces and allow surface functionalization with *N*-hydroxysuccinimide ester, which is highly reactive to the -NH_2_ groups of antibody. Choi et al. suggested an ITO polymer-modified polyvinyl-imidazole electrode with Ni (II) ions for the detection of hippuric acid (the toluene metabolite) [126]. Since the Ni (II) ions are highly reactive to the imidazole function, various Ni (II) ions bind to the polymer, thereby facilitating electron transfer to the electrode.

Particularly, a combination of the nanocomposites with conducting polymer and metal nanoparticles has also received considerable attention due to the combining excellent electrochemical and optical properties as well as improved catalytic stability [127]. Lu et al. investigated a novel nanocomposites film of conducting polymers including polypyrrole (PPy), SWCNT, and AuNPs [128]. These hybrid nanocomposites could not only provide strong catalytic activity toward the oxidation of epinephrine and uric acids but also exhibit excellent sensitivity, selectivity and stability of the biosensor.

## Conclusions

Several functional nanomaterials based on nanotechnology have provided an innovative solid substrate for a highly sensitive on-site analysis via signal amplification in electrochemical biosensors. We introduced various types of functional nanomaterials (carbon nanotubes, graphene, metallic, silica nanoparticles, nanowire, indium tin oxide, and organic polymers), which are commonly used for the construction of very effective electrode supporting matrices owing to their high electrical conductivity, huge surface area, etc. The surfaces can be functionalized with various organic groups (silanes, thiols and conducting polymers) for effective immobilization. We also addressed possible methods to increase the sensitivity of electrochemical signals by implementing labeling techniques applicable to a variety of electroactive nanotracers. These techniques can be utilized to the POC version of the electrochemical detection platform exemplified by lateral-flow immunoassay and microfluidic systems by means of deposition, patterning and electroactive conveying, both miniaturizing and improving analytical performance. Although the electrochemical biosensors have been shown to be suitable for high-performance analysis in diverse field applications, the matrix interference influencing the biomolecular interaction from real samples (blood, food, etc.) still remains the most critical issues that need to be solved for improving the analytical performances.

## Data Availability

Not applicable.
